# Supplementary material for: Binding Sites, Vibrations and Spin‐Lattice Relaxation Times in Europium(II)‐Based Metallofullerene Spin Qubits
Source: Chemistry. 2021 Aug 10;27(52):13242–8. doi: 10.1002/chem.202101922 (PMC8518920; doi:10.1002/chem.202101922)
Supplement: Supplementary file 1 — Supporting Information [file CHEM-27-13242-s001.pdf]

Chemistry–A European Journal

Supporting Information

Binding Sites, Vibrations and Spin-Lattice Relaxation Times in Europium(II)-Based Metallofullerene Spin Qubits

Ziqi Hu, Aman Ullah, Helena Prima-Garcia, Sang-Hyun Chin, Yuanyuan Wang, Juan Aragó, Zujin Shi,* Alejandro Gaita-Ariño,* and Eugenio Coronado*

Supporting Information

Contents

SI 1: Separations and characterizations

SI 2: DFT calculations

SI 3: EPR studies

SI 4: Spin-vibration coupling calculations

SI 1: Separations and characterizations

Figure S1.1 The multi-stage HPLC separation of Eu@C_{2n} (2n = 74-84) with toluene as eluent and 310nm detection wavelength. **a)** The first step: 20mm×250mm 5PYE column, flow rate: 15mL/min, the blue line shows enlarged spectrum; **b)** F3 separation: 20mm×250mm Buckyprep-M column, 15mL/min; **c)** F3-1 separation: 20mm×250mm Buckyprep column (recycle), 15mL/min; **d)** F3-1-1 separation: 10mm×250mm 5PBB column (recycle), 4mL/min. Numbering of isomers is according to order of retention time.

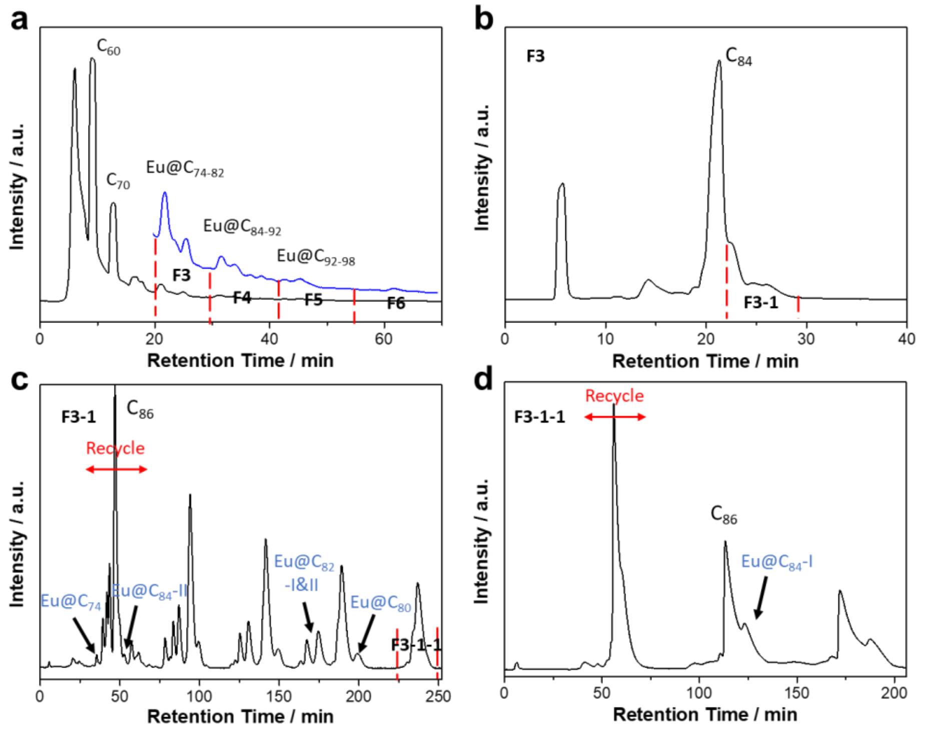

Figure S1.2 The multi-stage HPLC separation of Eu@C_{90} with toluene as eluent and 310nm detection wavelength. **a)** F4 separation: 20mm×250mm 5PBB column, 15mL/min; **b)** F4-1 separation: 20mm×250mm Buckyprep column (recycle), 15mL/min.

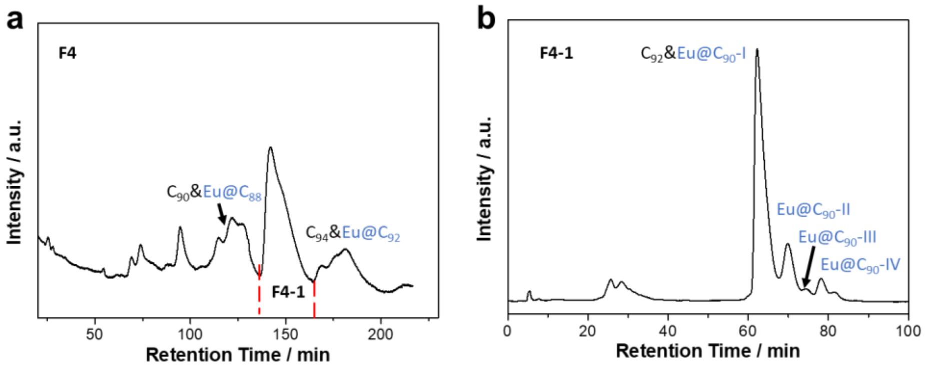

Figure S1.3 Normalized LD-TOF mass spectra of Eu@C_{2n}. The insets show that the isotopic distribution of experiments match with the calculated results, verifying the composition of isolated compounds. The spectra are vertically shifted for clarity.

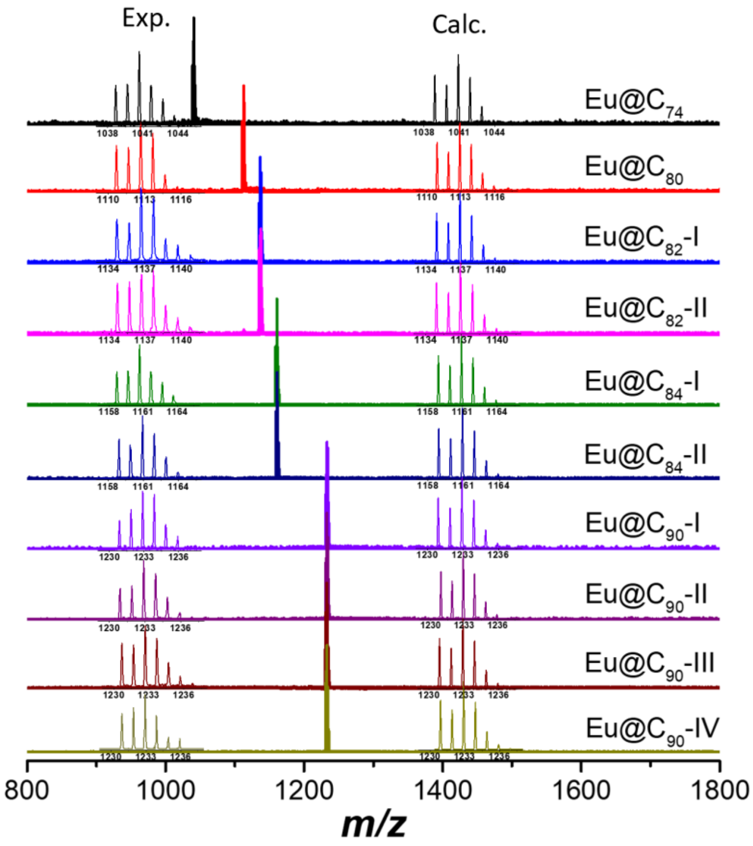

Figure S1.4 Normalized Vis-NIR absorption spectra of Eu@C_{2n}. Spectra are vertically shifted for clarity.

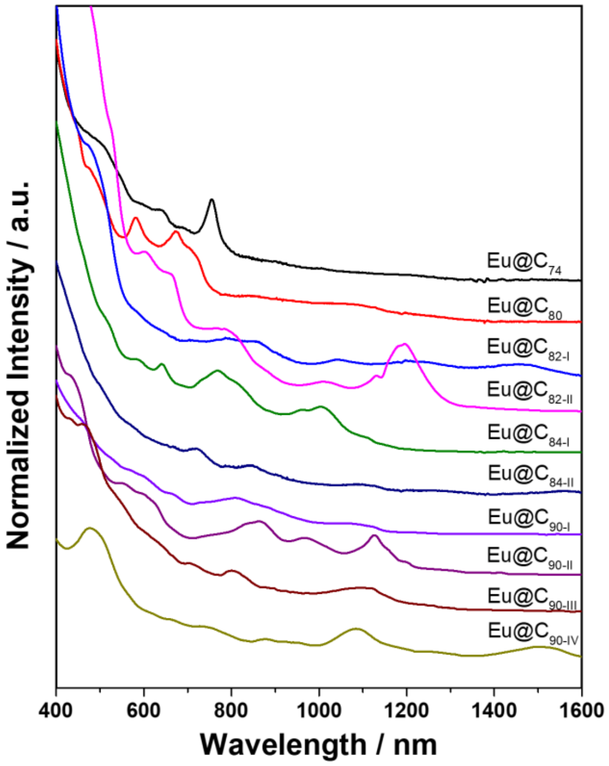

SI 2: DFT calculations

Figure S2.1 Side views (upper) and top views (down) of the optimized structures with hexagon binding motif (highlighted in red): **a)** Eu@C_{82}-C_2(5), **b)** Eu@C_{82}-C_{2v}(9), **c)** Eu@C_{84}-C_2(13), **d)** Eu@C_{90}-C_2(40), **e)** Eu@C_{90}-C_2(42) and **f)** Eu@C_{92}-C_1(42).

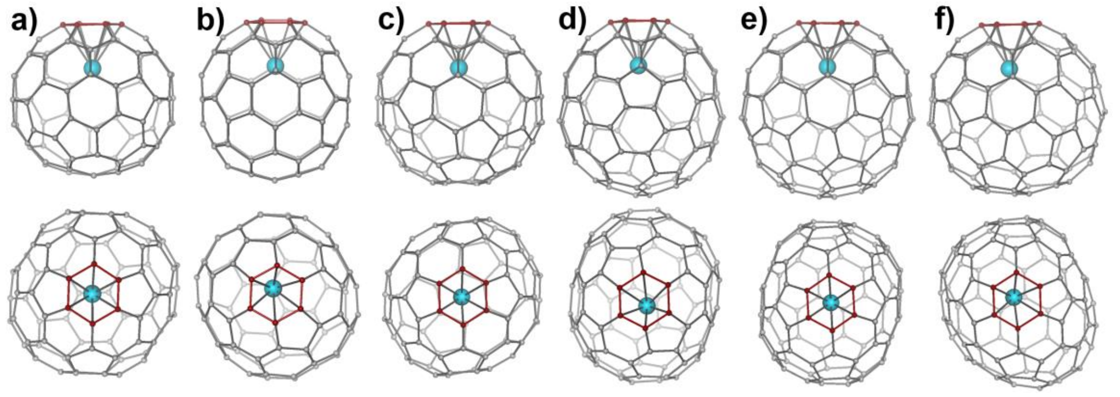

Figure S2.2 Side views (upper) and top views (down) of the optimized structures with pyracylene binding motif (highlighted in red): **a)** Eu@C_{74}-D_{3h}(5), **b)** Eu@C_{80}-C_{2v}(3), **c)** Eu@C_{84}-C_2(11), **d)** Eu@C_{90}-C_2(45) and **e)** Eu@C_{90}-C_{2v}(46).

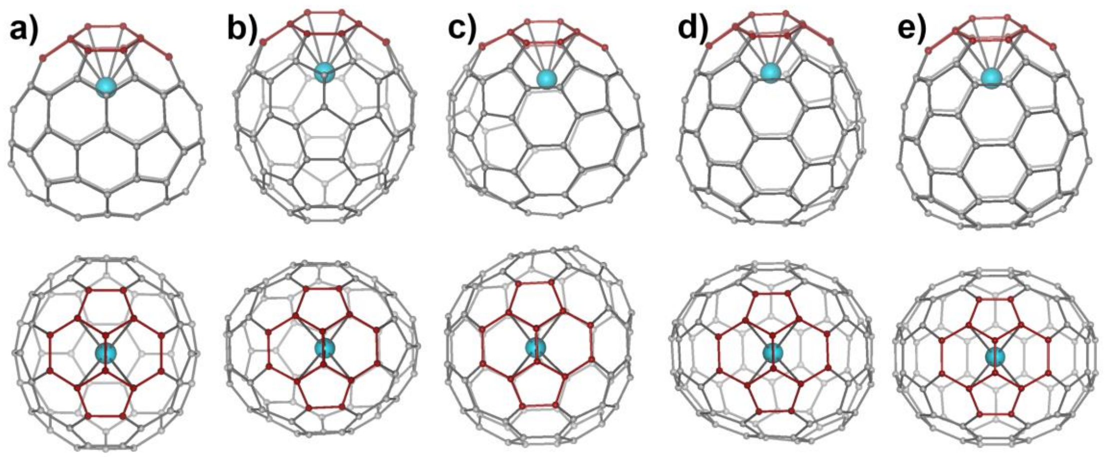

Figure S2.3 Side views (upper) and top views (down) of the optimized **a)** Eu@C_{82}-C_s(6), **b)** Eu@C_{82}-C_{3v}(7), **c)** Eu@C_{92}-C_s(24) and **d)** Eu@C_{94}-C_{3v}(134) with acephenalene binding motifs (highlighted in red).

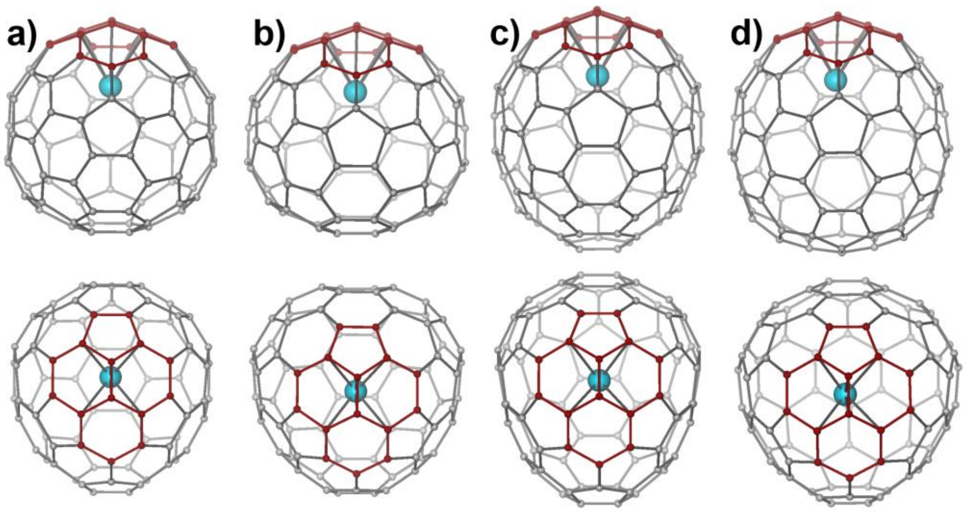

Figure S2.4 Side views (upper) and top views (down) of the optimized **a)** Eu@C_{76}-C_{2v}(19138) and **b)** Eu@C_{76}-C_1(17459) with fused-pentagon motifs (highlighted in red).

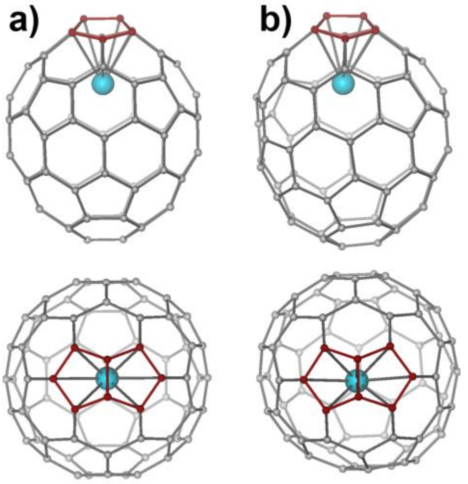

Figure S2.5 Correlations between averaged p-orbital axis vector (POAV), averaged frequencies $\overline{\nu_N}$ (N = 1-3) of the three metal-based vibrations and metal-cage delocalization indices δ(Eu, cage). The yellow lines indicate linear fittings with goodness-of-fit parameters R^2 being 0.94 **(a)**, 0.81 **(b)** and 0.89 **(c)**. The labelling is according to four different binding motifs as fused-pentagon (blue), pyracylene (red), acephenalene (green) and hexagon (black).

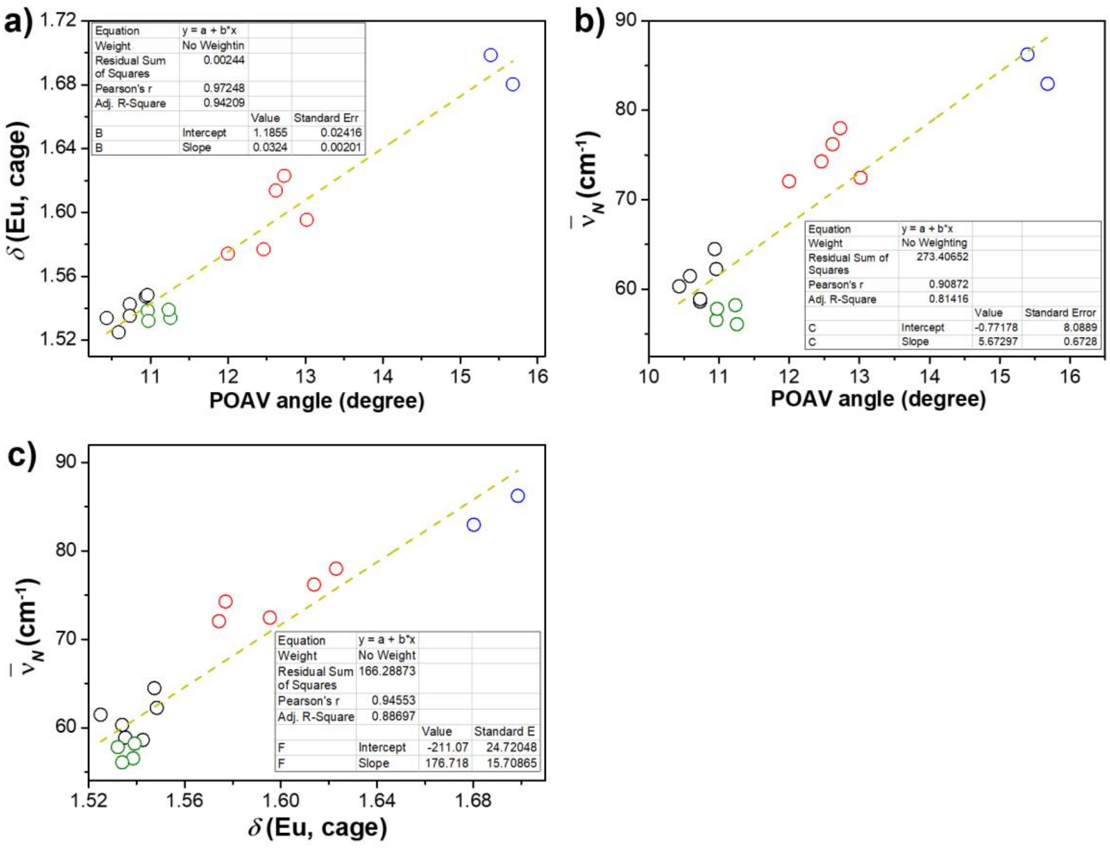

Figure S2.6 Pictures of the first 20 vibrational modes (modes 1-20 from upper left to down right) of Eu@C_{74} (**3**) including displacement vectors (blue arrows). The displacement vector of Eu atom of mode 1 is perpendicular to the paper plane.

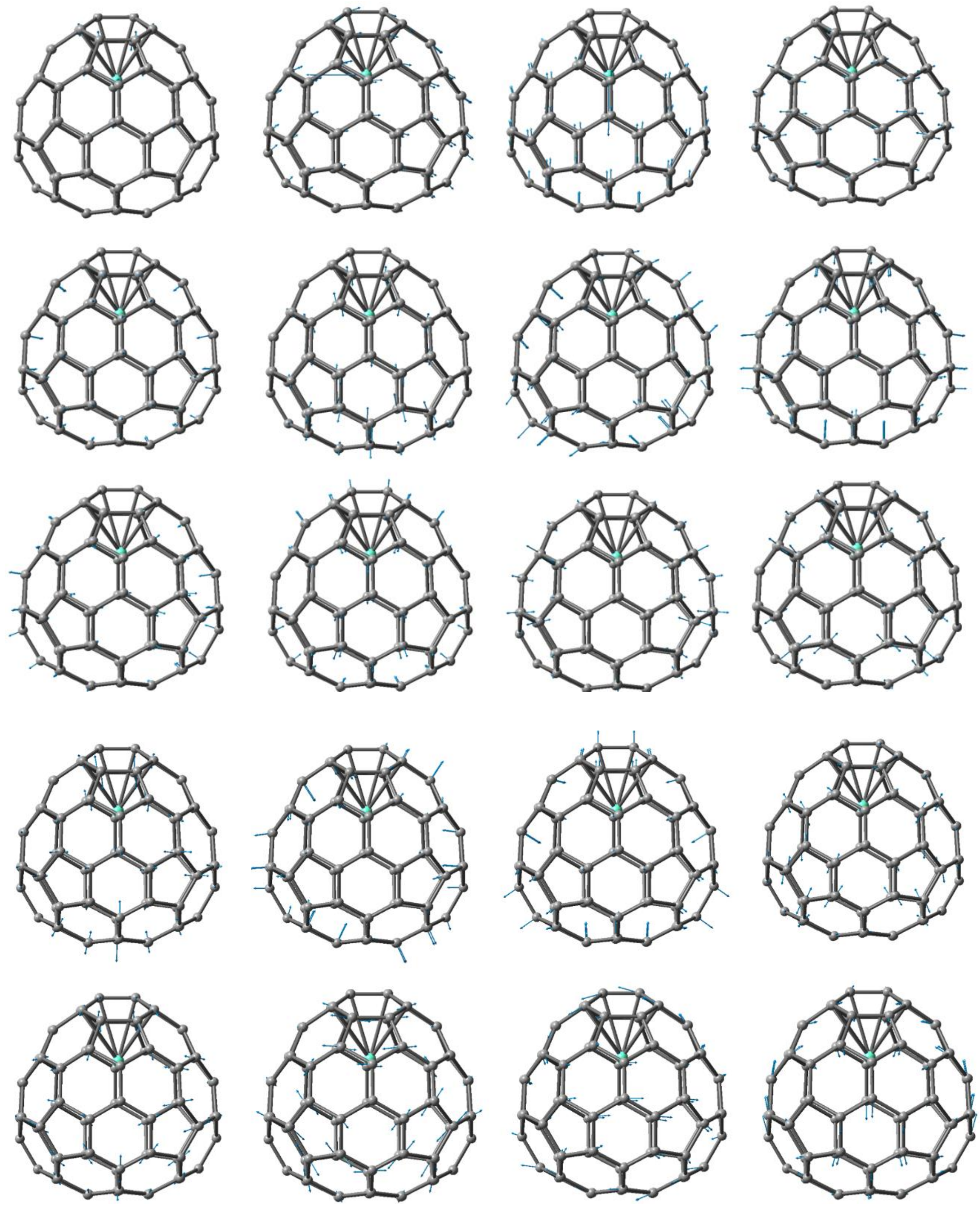

2-1. Metal binding sites

Let us discuss in detail our key assumption of a unique binding site for each EMF, which can be divided into two further assumptions: first, that there is a preferential binding site, and second, that the binding site is indeed maintained during the experiments. On the question of the preferential binding site, there is experimental and theoretical evidence to support our approximation. Indeed, among the various Eu@C_{2n} molecules we have synthesized, we carefully chose four cage structures for the study of spin dynamics, in each of which the major metal site can be clearly determined by XRD analysis and DFT calculation.

Specifically, Eu@C_{74} (**3**) and Eu@C_{80} (**3'**) are chosen as examples for pyracylene binding site. For **3**, it is widely accepted that the metal is located along with the C_3 axis of the cage. Given that there are three identical pyracylene motifs due to D_{3h} cage symmetry, the total occupancy of the major metal position around this pyracylene binding site is 0.607 from its crystal structure and the optimization of structure considering the minor metal site (under hexagon motif) eventually results in the same pyracylene site; for **3'**, it is well-known that the metal is located under pyracylene motif that is away from the C_2 axis of the cage. The occupancy of Eu major site is 0.797. It is also notable that if there is a suitable strong binding site such as pyracylene and fused pentagons of a cage (usually with a small or medium cage size), the metal is preferentially fixed at the major site with predominant occupancy in crystal structure. For weaker binding sites like acephenalene and hexagon, the metal is usually freer to have different locations inside the cage. In the case of Eu@C_{82}-C_s (**2**), although no crystal structure has been reported, Sm analogue clearly shows all metal positions close to the acephenalene major site[8] and this is also confirmed by our structural optimization for Eu-based complex; in the case of Eu@C_{84}-C_2(13) (**1**), the two major sites have occupancies of 0.179 and 0.124, and both are under a hexagon. The occupancies of the rest minor sites are less than 0.07. The optimizations of the two major sites both lead to the same configuration with Eu resides under the hexagon along with C_2 axis of the cage.

On the question of whether the binding site is stable, in our model we are assuming that the metal is 'fixed' at certain binding sites during the spin-lattice relaxation. Indeed, the energy barrier between the most stable and other Eu binding sites is calculated to be more than 5.0 kcal/mol of Eu@C_{2n} at B3LYP/6-31G(d)/MWB28 level, indicating that it is reasonable to consider that at low temperatures (< 20 K) a movement between binding sites will be thermally unfeasible. Nevertheless, there are indications that at least at high temperatures the Eu atom has certain mobility inside the cage as seen from the crystallographic data recorded at 100 K of various Eu@C_{2n}, displaying some disorder. It is reasonable that, depending on the dynamics of the cooling process, some ions will be trapped in a binding site corresponding to a local minimum different from the absolute minimum. While this is certainly a limitation of our model, we can safely assume the majority of the Eu ions will be in the real minimum and that the macroscopically observed relaxation times will be related to the binding site with the minimum energy.

Table S1 Computed structural information, vibrational frequencies and selected bonding parameters for Eu@C_{2n} compounds.

compound[a]	cage isomer	θ_p / degree[c]	ν / cm^{-1}[d]	δ(Eu, cage)[e]	BO(Eu, cage)[f]	Ref (M@C_{2n})[g]
Eu@C_{76}-C_{2v}[b]	C_{2v}(19138)	15.4	86.2	1.70	2.06	1(Eu)
Eu@C_{76}-C_1[b]	C_1(17459)	15.7	83.0	1.68	2.08	2(Sm)[h]
Eu@C_{74}	D_{3h}(5)	13.0	72.4	1.60	2.00	1(Eu)
Eu@C_{80}	C_{2v}(3)	12.4	74.3	1.58	1.97	1(Eu)
Eu@C_{84}-II	C_2(11)	12.0	72.1	1.57	1.94	3(Eu)[i]
Eu@C_{90}-III	C_{2v}(46)	12.7	76.2	1.62	1.99	4(Sm)
Eu@C_{90}-IV	C_2(45)	12.6	78.0	1.61	1.98	4(Sm)
Eu@C_{82}-II	C_2(5)	10.7	58.6	1.54	1.92	5(Eu)[i]
Eu@C_{82}-C_{2v}[b]	C_{2v}(9)	10.4	60.3	1.53	1.91	6(Sm)
Eu@C_{84}-I	C_2(13)	10.7	58.9	1.54	1.92	3(Eu)
Eu@C_{90}-I	C_2(40)	10.6	61.5	1.52	1.92	4(Sm)
Eu@C_{90}-II	C_2(42)	10.9	64.5	1.55	1.93	4(Sm)
Eu@C_{92}-C_1[b]	C_1(42)	11.0	62.2	1.55	1.94	7(Sm)
Eu@C_{82}-I	C_s(6)	11.0	56.5	1.54	1.91	8(Sm)
Eu@C_{82}-C_{3v}[b]	C_{3v}(7)	11.2	56.1	1.53	1.92	8(Sm)
Eu@C_{92}[b]	C_s(24)	11.2	58.2	1.54	1.93	7(Sm)
Eu@C_{94}[b]	C_{3v}(134)	11.0	57.8	1.53	1.92	7(Sm)

[a]Compounds are listed in different colors according to four binding motifs: fused pentagons (blue), pyracylene (red), hexagon (black) and acephenalene (green). [b]Compounds not obtained in this work. [c]Averaged POAV angle of hexagon is determined as the average value of the six carbon atoms since each of them coordinate with Eu^{2+} equally. The other three structures adopt the average of the two nearest carbon atoms (a C-C bond where Eu^{2+} resides). [d]Averaged frequency of the three Eu-cage vibrations along *xyz*-directions. [e]Delocalization index of Eu atom with cage, determined as the sum of all δ(Eu, C) values. [f]Mayer bond order of Eu atom with cage, determined as the sum of all BO(Eu, C) values. [g]References in which the crystal structures of M@C_{2n} (M = Eu or Sm) are used for optimizations of corresponding Eu@C_{2n} molecules. [h]Only DFT predicted structure is available. [i]The most stable geometries are calculated to show the minor metal sites (second largest occupancies) in the crystal structures at 100 K. All the other optimizations of compounds lead to the major metal sites in the corresponding crystals.

Table S2 Computed frequencies (v) and reduced masses (m in chemical atomic mass unit as u.m.a.q.) of the first 20 vibrational modes of Eu@C_{74} (**3**). The first three modes feature large reduced masses, indicative of the metal-dependent movements. The rest of them show reduced masses close to 12 u.m.a.q., suggesting that these modes are cage dependent.

**Mode**	**1**	**2**	**3**	**4**	**5**
v / cm^{-1}	44.6698	48.0083	124.2905	225.0935	227.9400
m / u.m.a.q.	48.4232	47.9202	48.9324	12.0000	12.0343
**Mode**	**6**	**7**	**8**	**9**	**10**
v / cm^{-1}	228.8824	239.4449	243.6319	295.6684	302.5454
m / u.m.a.q.	12.0088	12.0534	12.1911	12.0008	12.0333
**Mode**	**11**	**12**	**13**	**14**	**15**
v / cm^{-1}	305.8838	308.4076	313.2636	317.9848	327.3571
m / u.m.a.q.	12.0047	12.0000	12.0106	12.0107	12.0422
**Mode**	**16**	**17**	**18**	**19**	**20**
v / cm^{-1}	348.0165	349.3484	362.6041	366.2547	367.2146
m / u.m.a.q.	12.0000	12.0002	12.0000	12.0012	12.0006

Table S3 Computed frequencies (v) and reduced masses (m in chemical atomic mass unit as u.m.a.q.) of the first 20 vibrational modes of Eu@C_{76}-C_{2v} (**4**).

**Mode**	**1**	**2**	**3**	**4**	**5**
v / cm^{-1}	60.0878	69.6468	128.3551	214.8854	218.8218
m / u.m.a.q.	47.1005	47.2216	49.4219	12.0000	12.0002
**Mode**	**6**	**7**	**8**	**9**	**10**
v / cm^{-1}	238.3730	238.5245	254.2890	284.5491	288.7293
m / u.m.a.q.	12.0324	12.0382	12.2799	12.0001	12.0000
**Mode**	**11**	**12**	**13**	**14**	**15**
v / cm^{-1}	298.2109	301.7651	315.6163	322.5929	323.4960
m / u.m.a.q.	12.0028	12.0000	12.0292	12.0592	12.0370
**Mode**	**16**	**17**	**18**	**19**	**20**
v / cm^{-1}	335.8477	345.4773	347.4105	367.9376	372.0471
m / u.m.a.q.	12.0000	12.0000	12.0009	12.0004	12.0000

Table S4 Computed frequencies (v) and reduced masses (m in chemical atomic mass unit as u.m.a.q.) of the first 20 vibrational modes of Eu@C_{80} (**3'**).

**Mode**	**1**	**2**	**3**	**4**	**5**
v / cm^{-1}	44.0744	54.5408	124.2296	209.9742	211.3425
m / u.m.a.q.	50.2916	49.3248	48.3019	12.0052	12.0323
**Mode**	**6**	**7**	**8**	**9**	**10**
v / cm^{-1}	226.0051	236.7868	246.2104	278.4237	280.6624
m / u.m.a.q.	12.0112	12.0092	12.2753	12.0040	12.0001
**Mode**	**11**	**12**	**13**	**14**	**15**
v / cm^{-1}	287.4314	295.0851	300.1381	315.0750	318.2296
m / u.m.a.q.	12.0212	12.0005	12.0155	12.0338	12.0494
**Mode**	**16**	**17**	**18**	**19**	**20**
v / cm^{-1}	327.4031	332.3409	348.2345	360.6661	362.3946
m / u.m.a.q.	12.0010	12.0000	12.0108	12.0016	12.0018

Table S5 Computed frequencies (v) and reduced masses (m in chemical atomic mass unit as u.m.a.q.) of the first 20 vibrational modes of Eu@C_{82}-C_s (**2**).

**Mode**	**1**	**2**	**3**	**4**	**5**
v / cm^{-1}	17.1335	25.4287	127.0512	212.7064	216.6156
m / u.m.a.q.	51.2401	51.5152	43.5150	12.0004	12.0423
**Mode**	**6**	**7**	**8**	**9**	**10**
v / cm^{-1}	222.2069	226.7913	233.5127	279.2843	279.7262
m / u.m.a.q.	12.0017	12.0036	12.4123	12.0004	12.0071
**Mode**	**11**	**12**	**13**	**14**	**15**
v / cm^{-1}	284.6843	292.4182	294.0394	296.4330	306.7298
m / u.m.a.q.	12.0009	12.0076	12.0038	12.0127	12.2181
**Mode**	**16**	**17**	**18**	**19**	**20**
v / cm^{-1}	336.1954	338.2143	340.4583	354.2809	354.7322
m / u.m.a.q.	12.0000	12.0000	12.0000	12.0009	12.0000

Table S6 Computed frequencies (v) and reduced masses (m in chemical atomic mass unit as u.m.a.q.) of the first 20 vibrational modes of Eu@C_{84}-C_2(13) (**1**).

**Mode**	**1**	**2**	**3**	**4**	**5**
v / cm^{-1}	14.5899	35.9896	126.0767	211.0710	212.1702
m / u.m.a.q.	51.6609	51.7751	43.7165	12.0027	12.0848
**Mode**	**6**	**7**	**8**	**9**	**10**
v / cm^{-1}	216.2052	222.9360	235.1855	274.7698	281.9020
m / u.m.a.q.	12.0006	12.0021	12.3471	12.0022	12.0024
**Mode**	**11**	**12**	**13**	**14**	**15**
v / cm^{-1}	282.1510	283.9050	289.0612	296.7128	303.6481
m / u.m.a.q.	12.0033	12.0369	12.0068	12.0046	12.2039
**Mode**	**16**	**17**	**18**	**19**	**20**
v / cm^{-1}	324.2981	332.5726	337.5730	344.8727	350.6549
m / u.m.a.q.	12.0024	12.0003	12.0040	12.0000	12.0032

SI 3: EPR studies

Figure S3.1 a) X-band cw-EPR spectrum recorded at 4 K of Eu@C_{80} (**3'**). The simulation lines are plotted in blue with the ZFS parameters of D = 0.275 cm^{-1} and E = 0.0025 cm^{-1}. An isotropic g factor of g_{iso} = 1.99 and a ZFS strain (Str_D = 0.006 cm^{-1} and Str_E = 0.003 cm^{-1}) accounting for inhomogeneous broadening are applied. **b)** Echo-detected field-sweep (EDFS) spectrum of Eu@C_{80} (**3'**) at 3.3 K. The blue line represents the simulation of absorption spectrum based on the same set of parameters determined in the cw-EPR fitting. The inset symbols indicate the main transitions at different magnetic fields, which are further investigated for the spin dynamics.

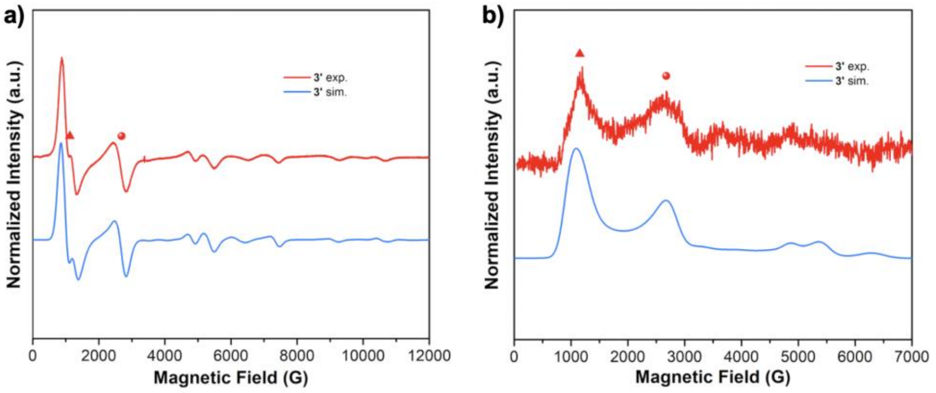

Figure S3.2 Zeeman splitting for the S = 7/2 spin of three samples illustrated in **Figure 2** when the magnetic field is parallel to **a)** x- and **b)** y-axis of the ZFS tensor. The blue circles show the positions of the transitions with 9.75 GHz microwave photons.

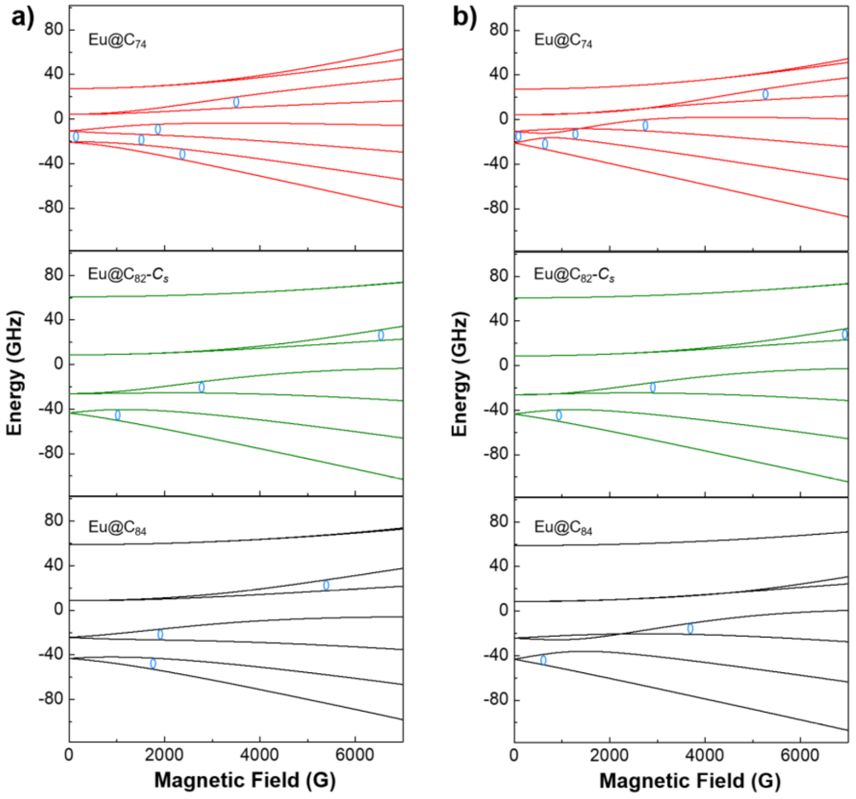

Figure S3.3 Echo decays of Eu@C_{74} (**3**) in CS_2 solution at different temperatures and magnetic fields.

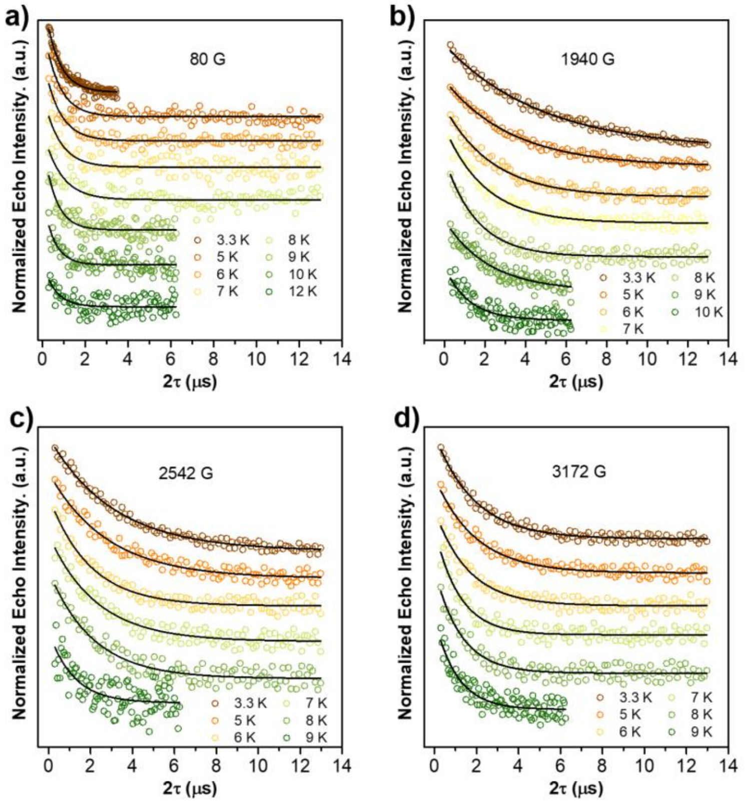

Figure S3.4 T_1 curves of Eu@C_{74} (**3**) in CS_2 solution at different temperatures and magnetic fields.

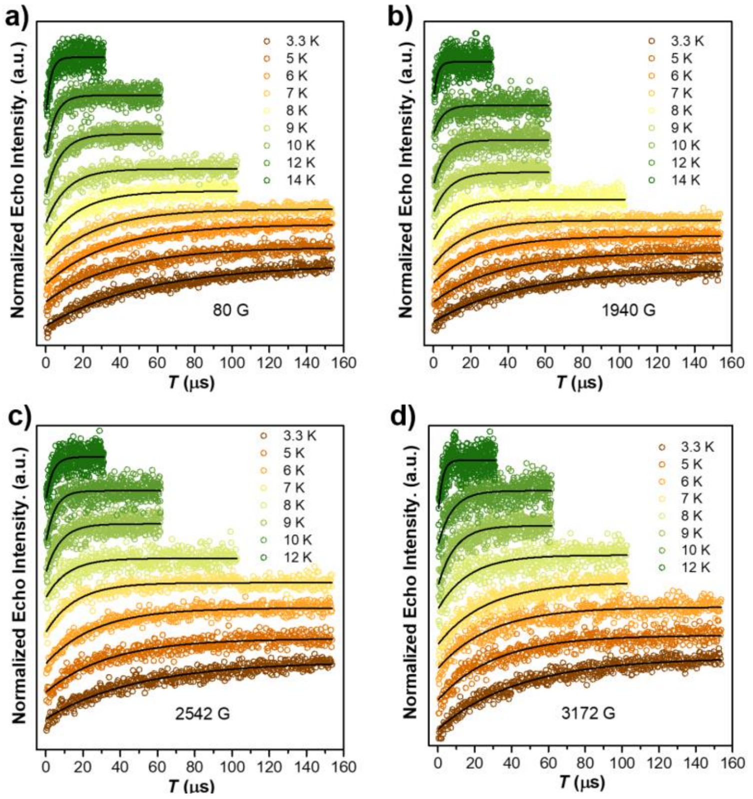

Figure S3.5 Echo decays of Eu@C_{82}-C_s (**2**) in CS_2 solution at different magnetic fields at 3.3 K (solid) and 5 K (open).

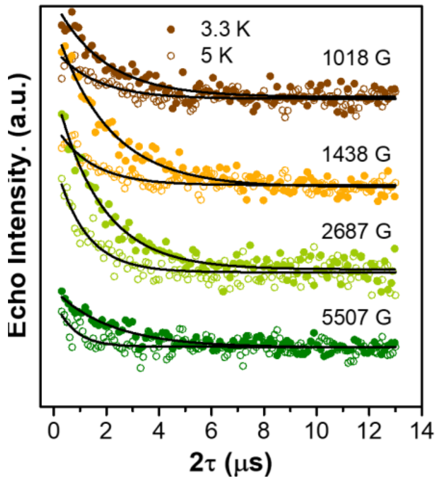

Figure S3.6 T_1 curves of Eu@C_{82}-C_s (**2**) in CS_2 solution at different temperatures and magnetic fields.

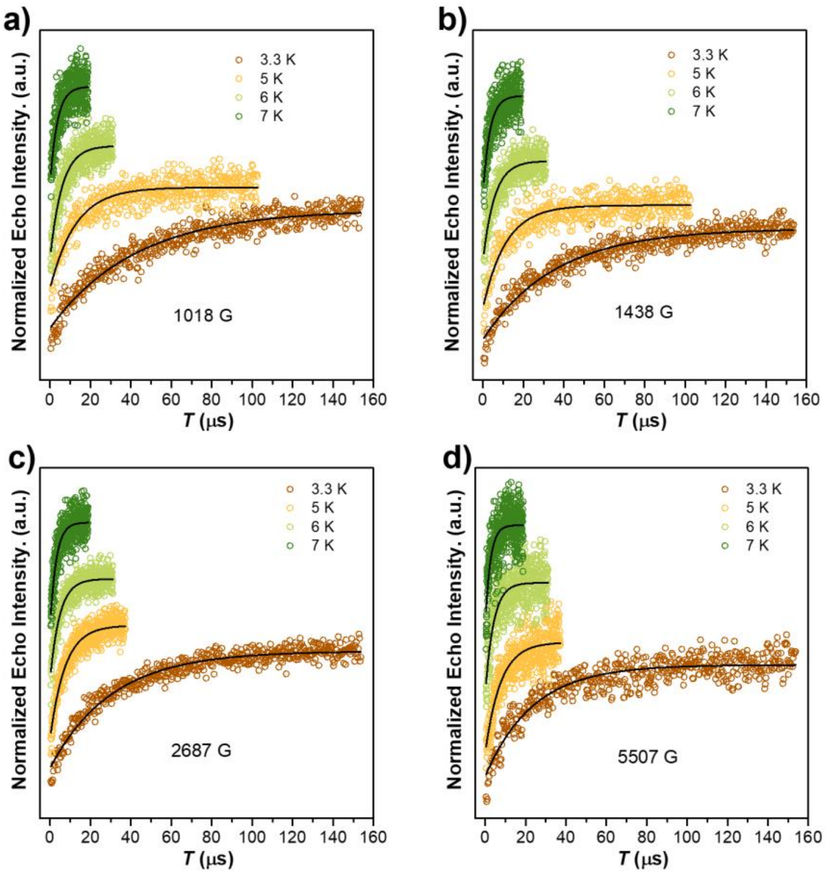

Figure S3.7 T_1 curves of Eu@C_{80} (**3'**) in CS_2 solution at different temperatures at **a)** 1159 G and **b)** 2547 G; echo decay curves at different temperatures at **c)** 1159 G and **d)** 2547 G.

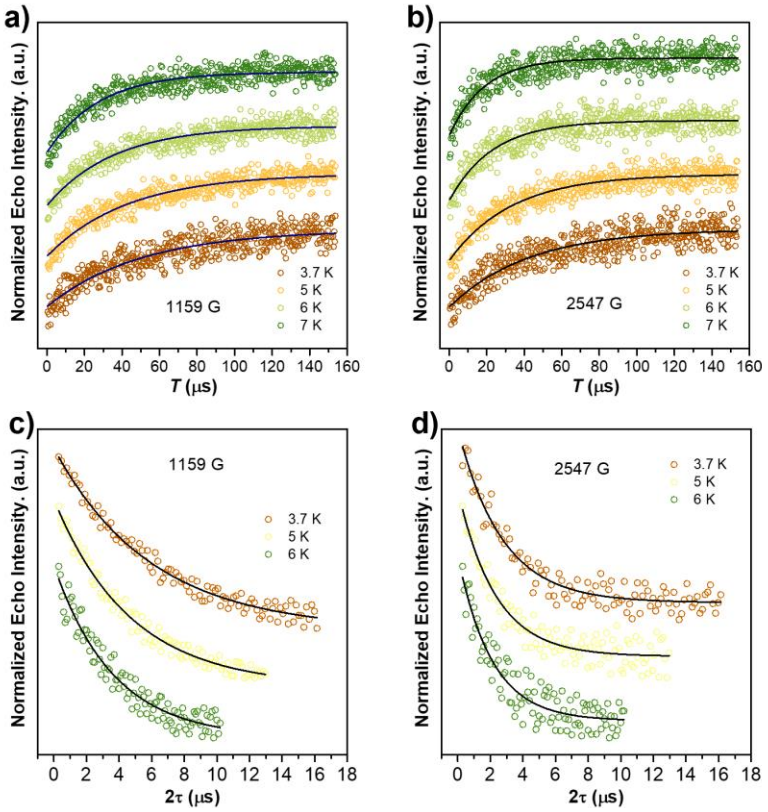

Figure S3.8 Echo decay curves of Eu@C_{84} (**1**) in CS_2 solution at different temperatures at **a)** 1464 G and **b)** 1800 G; T_1 curves at different temperatures at **c)** 1464 G and **d)** 1800 G.

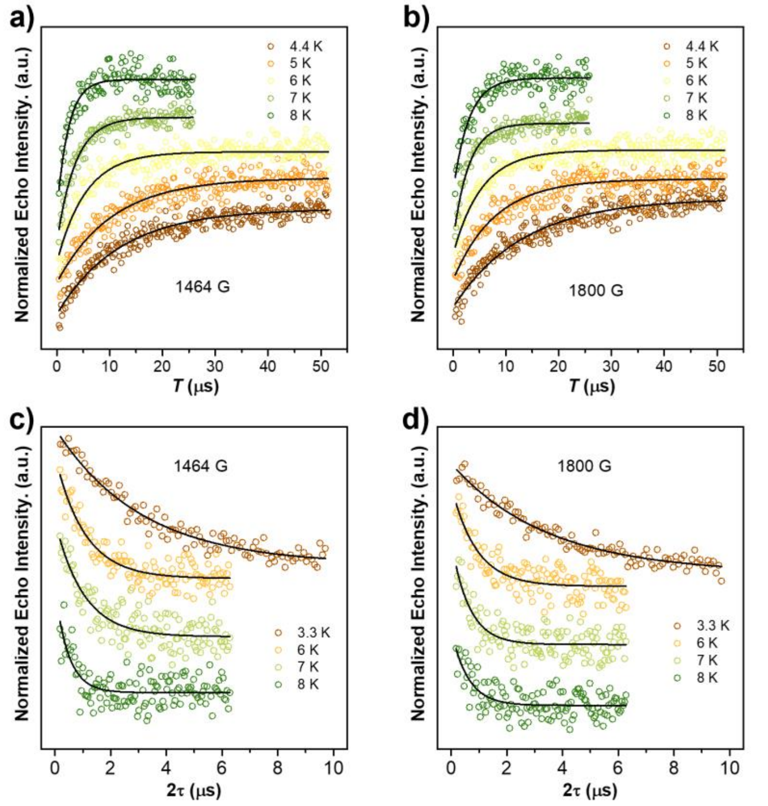

Figure S3.9 Echo decays of Eu@C_{74} (**3**) in d^8-toluene solution at different magnetic fields at 3.3 K. The oscillations indicate the nuclear-spin modulation of the electronic spin. The maximum phase memory time T_m is 1.2 μs at 1940 G.

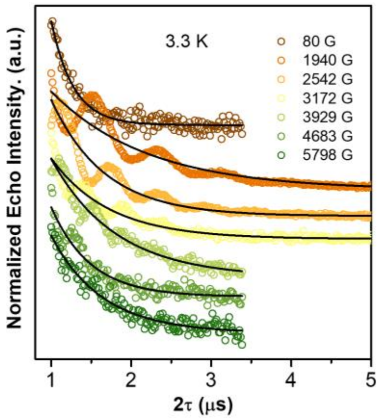

Figure S3.10 Echo decays of Eu@C_{74} (**3**) in d^8-toluene solution at selected magnetic fields at different temperatures. The different start points are due to different initial τ values.

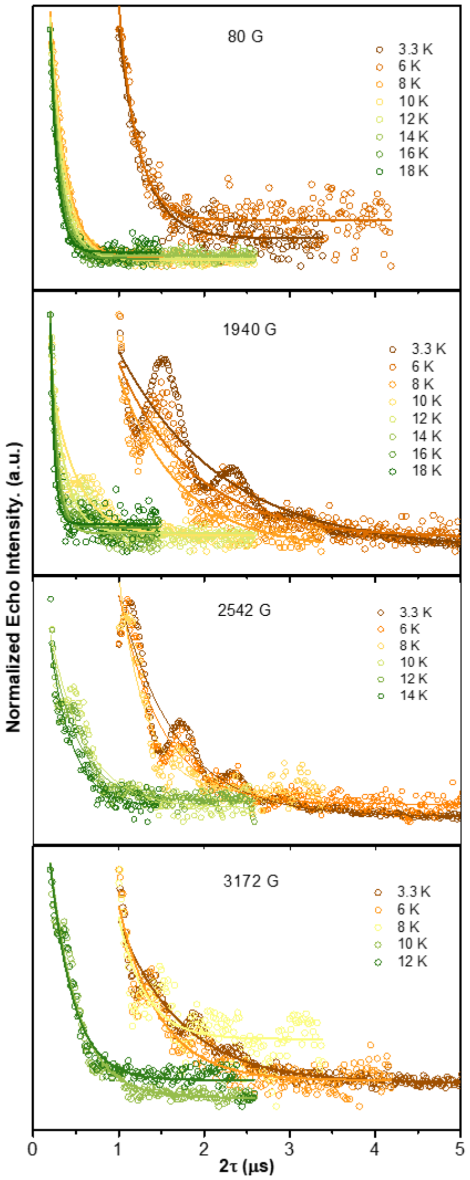

Figure S3.11 T_1 curves of Eu@C_{74} (**3**) in d^8-toluene solution at different temperatures and magnetic fields.

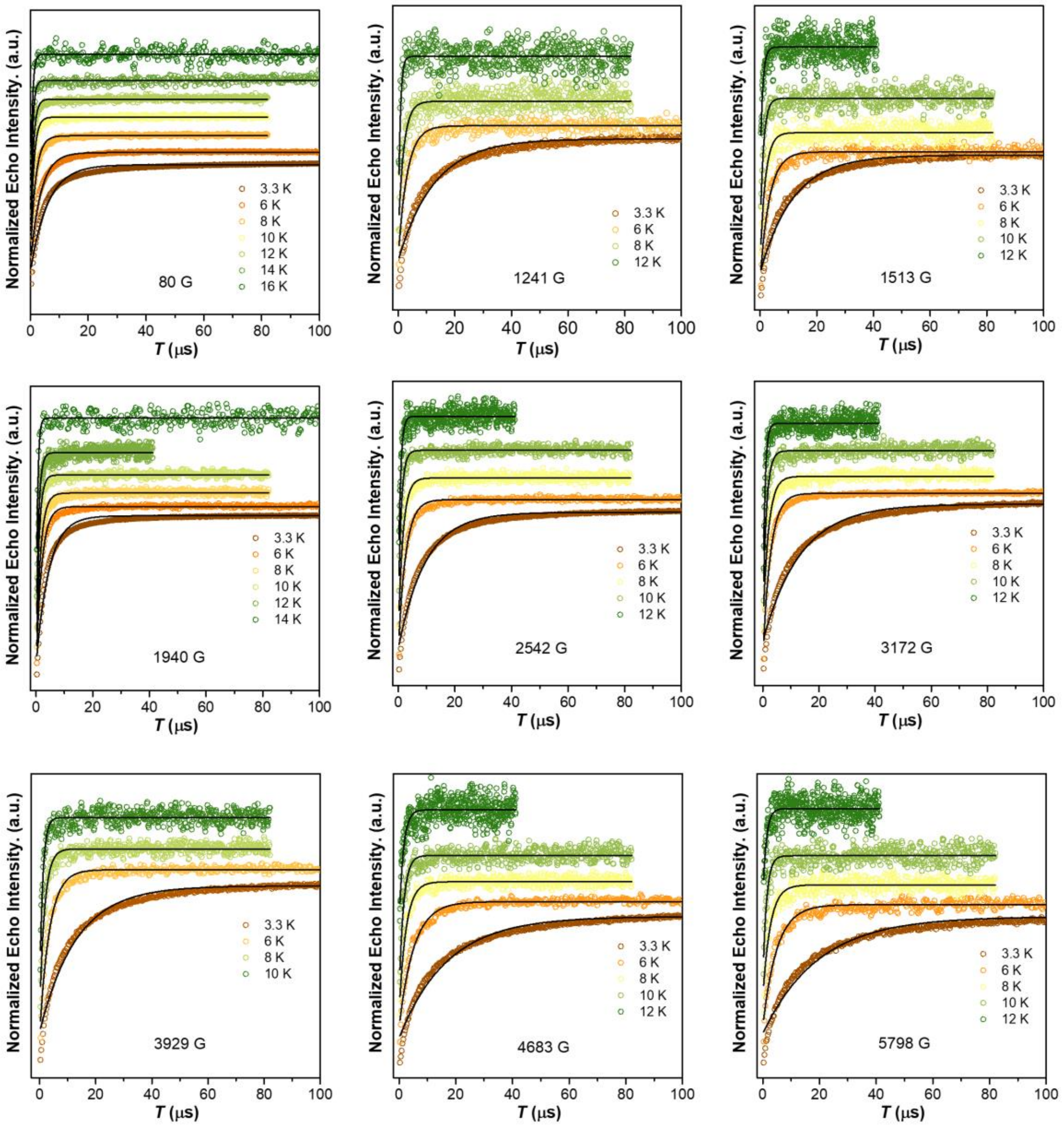

Figure S3.12 Echo decays of Eu@C_{82}-C_s (**2**) in d^8-toluene solution at different temperatures and magnetic fields. The oscillations indicate the nuclear-spin modulation of the electronic spin. The maximum phase memory time T_m is 1.7 μs at 2687 G at 3.3 K.

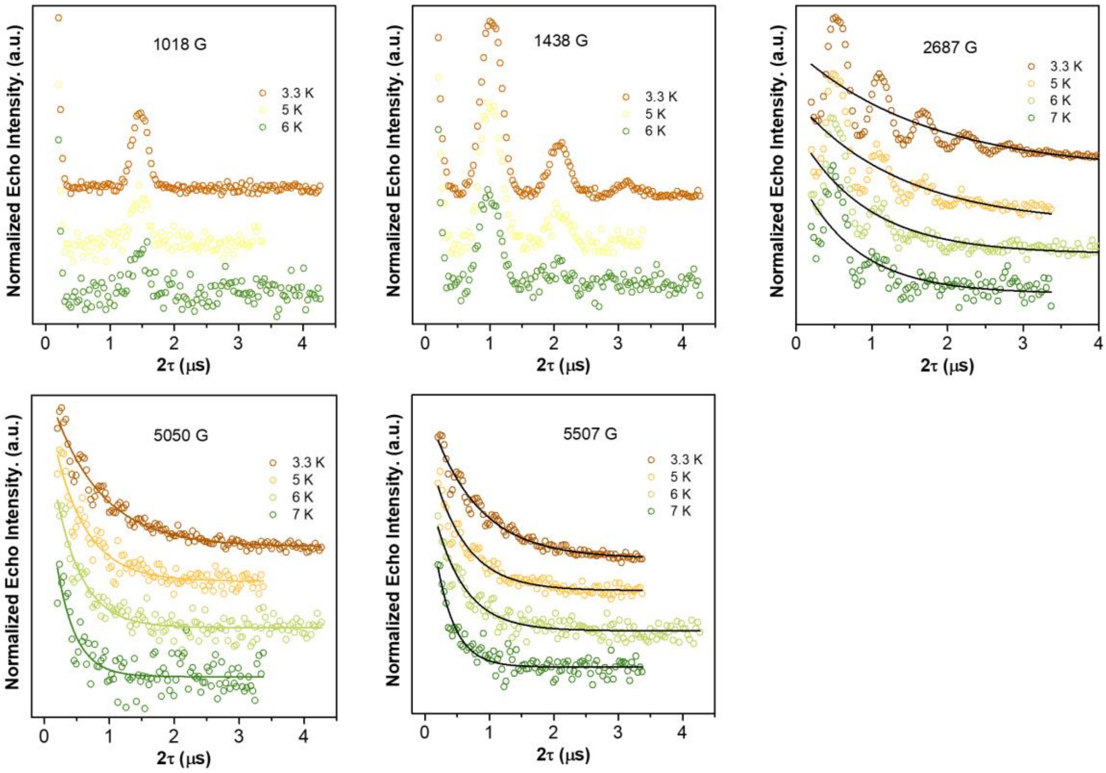

Figure S3.13 T_1 curves of Eu@C_{82}-C_s (**2**) in d^8-toluene solution at different temperatures and magnetic fields.

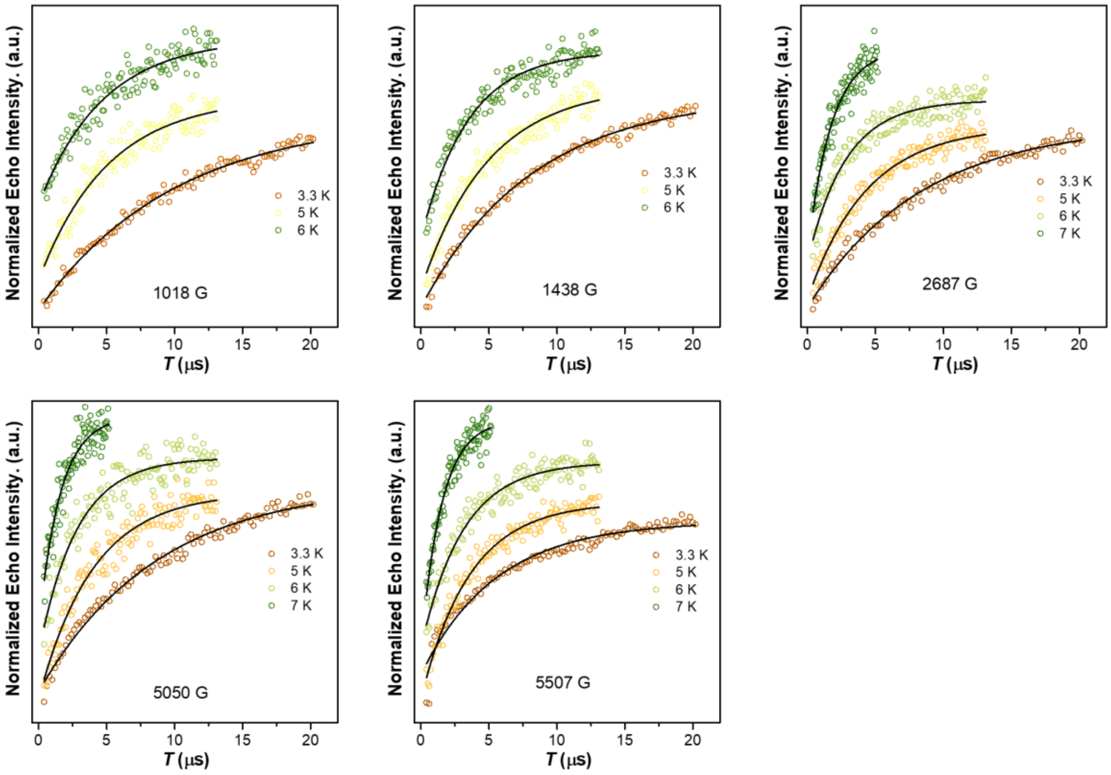

Figure S3.14 Different fits of T_1-temperature dependence of Eu@C_{2n} in CS_2 solution. The combination of possible relaxation mechanisms is described as $T_1^{-1} = A_{Dir}T + CT^n + \tau_0^{-1}\exp(-U^{\mathrm{eff}}/T)$, where direct, Raman and Orbach processes are taken into consideration, respectively. The Orbach fits (dashed lines, left panel) are probed for rigid (red, above 7 K, with τ_0^{-1} = 0.4(1) μs^{-1} and U^{eff} = 19.4(16) cm^{-1}) and soft (black, with τ_0^{-1} = 0.50(6) μs^{-1} and U^{eff} = 9.6(4) cm^{-1}) groups. These fits are lesser overlapped with experimental results than Raman process shows (**Figure 3a**). For the whole temperature range of T_1 in Eu@C_{74} (**3**) and Eu@C_{80} (**3'**), a total fit (black line, right panel) is employed combining Raman and direct mechanisms, with A_{Dir} = 0.0056(3) $\mu s^{-1}K^{-1}$, C = 1.7(11) × 10^{-7} $\mu s^{-1}K^{-n}$ and n = 5.7(8).

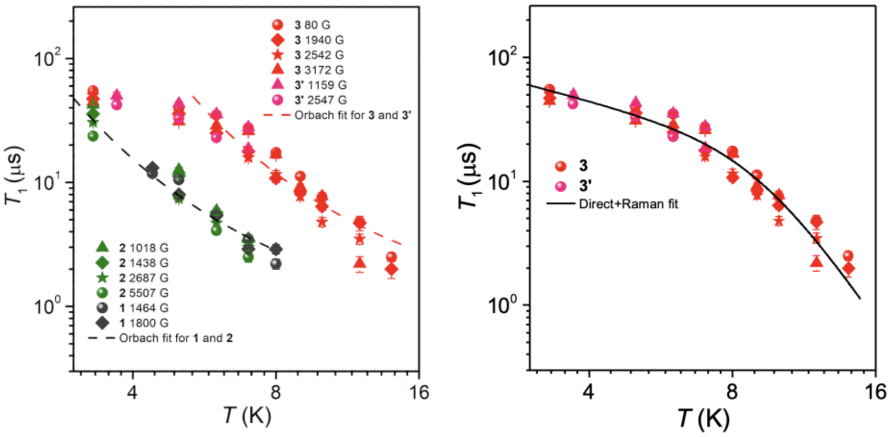

Figure S3.15 Left panel: temperature dependences of T_1 and T_m of **2** and **3** in d^8-toluene solution at different magnetic fields. Right panel: Rabi oscillations of **3** in d^8-toluene solution at 3172 G and 3.3 K with different attenuation powers. The inset shows the microwave pulse sequence applied.

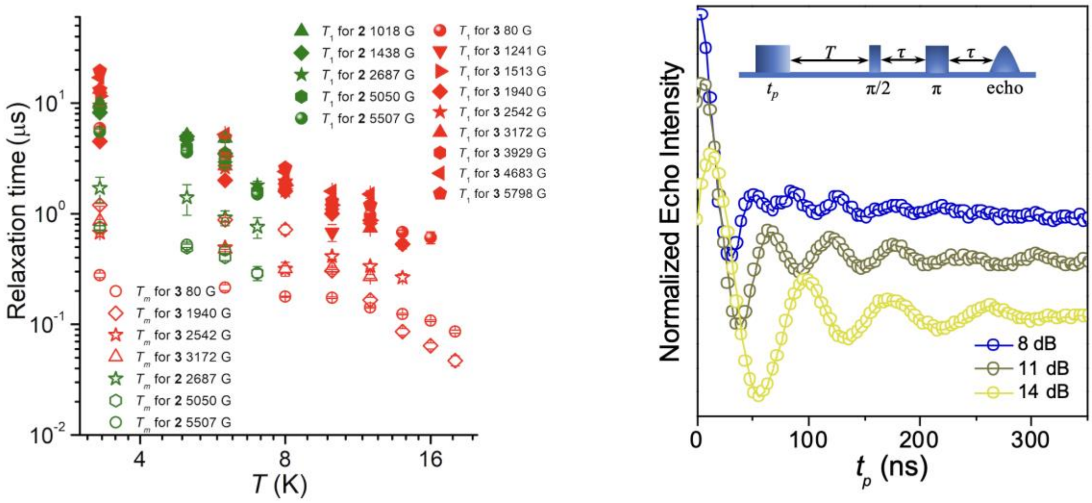

SI 4: Spin-vibration coupling calculations

4-1. Generation of the distorted geometries

The distorted geometries were obtained by applying the normalized displacement vector $\overrightarrow{R_k}(x_i, y_i, z_i)$ (shortly as $\overrightarrow{R_k}$) of each vibrational mode k to the optimized molecular coordinates vector $\overrightarrow{R_{eq}}(x_i, y_i, z_i)$ (shortly as $\overrightarrow{R_{eq}}$), leading to $\overrightarrow{R_{dist}} = \overrightarrow{R_{eq}} \pm Q_k \cdot \overrightarrow{R_k}$, where $\overrightarrow{R_{dist}}$ and Q_k are the distorted molecular geometry and the displacement along the mode *k*, respectively. The equilibrium geometry is recovered when $Q_k = 0$ for every vibrational mode. To keep the distorted geometries in the harmonic regime, we chose the upper limit of distortion Q_{k0} as the zero-point vibration level *n* = 0 according to harmonic oscillator approximation

$$Q_{k0} = \sqrt{h\nu_k/k} \ , \quad (1)$$

where ν_k is the vibrational frequency and *k* is the force constant of the vibration that can be obtained from Gaussian output file.

Notably, modes 1-3 are the only ones which present significant displacement vectors for Eu^{2+} (x_{Eu}, y_{Eu}, z_{Eu}): (0.51, 0.00, 0.00), (0.00, 0.50, 0.00) and (0.00, 0.00, 0.51) of Eu@C_{74} (**3**) as an example. In contrast, in all other modes only the carbon atoms move significantly, with the components for the displacement vector for Eu^{2+} being in all cases < 0.04. In addition, thanks to the rigidity of the cage, only minor distortions can possibly occur in the coordination environment purely due to carbon movements. Instead, the primary factor to change the coordination environment is the movement of the Eu^{2+} ion. We therefore assumed that only in modes 1-3 the coordination sphere is significantly altered, and thus these are the only ones where significant vibrational coupling can occur.

4-2. Spin-vibration coupling (SVC)

The effective Hamiltonian of the spin-phonon coupled system is composed of the spin ($\hat{H}_S$), the phonons ($\hat{H}_{vib}$) and the spin-vibration coupling ($\hat{H}_{S-vib.}$):

$$\hat{H}_{eff} = \hat{H}_S \otimes \mathbb{I}_{vib} + \mathbb{I}_S \otimes \hat{H}_{vib} + \hat{H}_{S-vib}, \quad (2)$$

where $\mathbb{I}_{vib}$ and $\mathbb{I}_S$ are identity matrices. The spin Hamiltonian $\hat{H}_S$ for an *S* = 7/2 spin without a magnetic field is defined as:

$$\hat{H}_S = D[\hat{S}_z^2 - \frac{1}{3}S(S+1)] + E(\hat{S}_x^2 - \hat{S}_y^2). \quad (3)$$

The vibrational Hamiltonian $\hat{H}_{vib}$ is assumed harmonic:

$$\hat{H}_{vib} = \hbar\nu\left(n + \frac{1}{2}\right). \quad (4)$$

$\hat{H}_S$ and $\hat{H}_{vib}$ are defined in $|M_S, n\rangle$ basis. In weak coupling regime, vibrational states are unaffected by spin energy levels, the matrix element for $\hat{H}_{S-vib}$ can be written as:

$$\langle M_S', n \pm 1 | \hat{H}_{S-vib.} | M_S, n \rangle$$
$$= \left(\left(\frac{\partial D}{\partial Q_k}\right)_{eq} [\hat{S}_z^2 - \frac{1}{3}S(S+1)] + \left(\frac{\partial E}{\partial Q_k}\right)_{eq} \left(\hat{S}_x{}^2 - \hat{S}_y{}^2\right) \right) \otimes \langle n \pm 1 | \hat{Q} | n \rangle, \quad (5)$$

where Q_k represents the displacement the kth-mode vibration causes to the molecule, $\left(\frac{\partial D}{\partial Q_k}\right)_{eq}$ and $\left(\frac{\partial E}{\partial Q_k}\right)_{eq}$ are spin-vibration coupling terms. For a single excitation, we can write:

$$\langle n + 1 | \hat{Q} | n \rangle = \begin{pmatrix} 0 & \frac{1}{\sqrt{2}} \\ \frac{1}{\sqrt{2}} & 0 \end{pmatrix}. \quad (6)$$

The overall coupling strength for each vibrational mode can be defined as S_k, if the second-order crystal-field parameters B_2^0 and B_2^2 are presented[9]:

$$S_k = \sqrt{\frac{1}{3} \cdot \frac{1}{5} \left[\left| \left(\frac{\partial B_2^0}{\partial Q_k}\right)_{eq} \right|^2 + 2 \left| \left(\frac{\partial B_2^2}{\partial Q_k}\right)_{eq} \right|^2 \right]}. \quad (7)$$

Since we are using D-tensor ZFS to describe this spin system with relations of $D = 3B_2^0$ and $E = B_2^2$, the above equation can be rewritten as:

$$S_k = \sqrt{\frac{1}{3} \cdot \frac{1}{5} \left[\left| \frac{1}{3} \left(\frac{\partial D}{\partial Q_k}\right)_{eq} \right|^2 + 2 \left| \left(\frac{\partial E}{\partial Q_k}\right)_{eq} \right|^2 \right]}. \quad (8)$$

The overall SVC strength S_k at CASSCF level for the first three vibrational modes of **1**, **2** and **3** is listed in **Table S7**.

4-3. Acoustic phonon coupling

We have established the first step in the spin relaxation process, namely the spin-vibration coupling governing the transfer of energy between the spin subsystem and the local molecular vibrations. After this point, the energy dissipation towards the thermal bath proceeds via the lattice phonons. Thus, it is important to probe how the acoustic, "Debye-like" EMF deformations due to solvent lattice phonons interact with the low energy gas-phase vibrational modes of interest. The goal at this point is to evaluate the coupling between local vibrations and lattice phonons, and in particular to verify whether the vibrations we identify as critical with respect to their spin-vibration coupling experience a direct coupling to extended phonons, or whether some previous internal energy transfer is needed.

A full integration of the phonon spectrum is challenging in our case involving a frozen solvent. Our approach instead is generic and based on symmetry. We deal with idealized acoustic phonons, which can be *a*) either longitudinal or transverse, and with *b*) *x*, *y* or *z* vector of propagation. This can be easily translated into displacements for any given molecular geometry. In the equilibrium

geometry $\overrightarrow{R_{eq}}$, we set Eu center as the origin with the *xyz* axes coincident with the vibrational modes 1-3 illustrated in **Figure 1a**.

We first defined a normalized compression vector $\vec{P}$, which describes the compression of molecular coordinates $\overrightarrow{R_{eq}}$ along *x*-, *y*- or *z*-direction. For instance, *x*-direction compression is:

$$\vec{P}(x_i) = \frac{a \cdot \overrightarrow{R_{eq}}(x_i)}{|a \cdot \overrightarrow{R_{eq}}(x_i)|}, \tag{9}$$

where a is the compression coefficient ($0 < a < 1$). The coupling strength between acoustic phonon and each vibrational mode can be estimated as the scalar product of the compression vector $\vec{P}$ and the vibrational vector $\overrightarrow{R_k}$. We proceeded to define two idealized types of acoustic phonons. One is longitudinal phonon with an interaction of $\vec{P}$ and $\overrightarrow{R_k}$ along with the same *x*-, *y*- or *z*-direction. For instance, the estimated coupling strength (S) of the longitudinal *x* phonon is:

$$S_x = \sum_i \vec{P}(x_i) \cdot \overrightarrow{R_k}(x_i). \tag{10}$$

The other is transverse phonon with an interaction of $\vec{P}$ and $\overrightarrow{R_k}$ at different directions (*xy*, *yx*, *xz*, *zx*, *yz* and *zy*). For instance, the coupling strength of the transverse *xy* phonon is defined as:

$$S_{xy} = \sum_i \vec{P}(x_i) \cdot \overrightarrow{R_k}(y_i). \tag{11}$$

With these definitions, we are able to qualitatively estimate (*a*) which vibrational modes couple strongly with the acoustic phonons in the solid and (*b*) which kind of phonons couple the most to the three low energy vibrations of interest. Using **3** as an example, the calculated coupling strength of the first 20 vibrations are listed in **Table S8**. The results indicate that modes 1-8 show significant coupling with the acoustic phonons, while such coupling for modes 9-20 is negligible. Compared with modes 1-3, however, modes 4-8 (*a*) do not involve significant changes of the Eu^{2+} coordination sphere and (*b*) are much higher in energies (> 215 cm^{-1}) as discussed above as well as in the main text. Thus, we conclude that modes 1-3 are the dominant factors governing the spin relaxation process, given that they not only effectively facilitate acoustic phonon-bath relaxation but also couple the most to the spin sublevels. Further, a closer analysis of the coupling between acoustic phonons with modes 1-3 reveals that *a*) longitudinal phonon couples strongly with the vibrational mode 3, which shows *z*- displacement of Eu; *b*) transverse phonons couple strongly with the vibrational modes 1 and 2, which show *x*- and *y*- displacement of Eu, respectively.

Table S7. Calculated overall SVC strength S_k (in cm^{-1}) at CASSCF level for the first three vibrational modes of Eu@C_{2n} molecules **1**, **2** and **3**.

	Eu@C_{84}-C_2 (**1**)	Eu@C_{82}-C_s (**2**)	Eu@C_{74} (**3**)
Mode-1	1.19E-03	6.35E-03	2.05E-04
Mode-2	5.26E-04	3.31E-03	2.98E-04
Mode-3	1.14E-02	1.09E-02	8.49E-03

Table S8. Coupling strength between acoustic phonons (longitudinal and transverse) and the first 20 vibrational modes in Eu@C_{74} (**3**).

	Longitudinal phonon			Transverse phonon					
**Mode**	*x*	*y*	*z*	*xy*	*yx*	*xz*	*zx*	*yz*	*zy*
1	0.00	0.00	0.00	0.00	0.00	0.00	0.00	-0.32	-0.24
2	0.00	0.00	0.00	0.00	0.00	-0.37	-0.30	0.00	0.00
3	0.09	0.11	-0.69	0.00	0.00	-0.02	-0.01	0.00	0.00
4	0.00	0.00	0.00	0.65	0.68	0.00	0.00	0.00	0.00
5	-0.48	0.74	-0.18	0.00	0.00	0.01	0.01	-0.01	-0.01
6	-0.01	0.01	0.00	0.00	0.01	0.00	0.00	0.67	0.51
7	0.02	0.00	-0.02	0.00	0.00	0.65	0.53	0.00	0.00
8	0.61	0.13	-0.54	0.00	0.00	-0.02	-0.02	0.00	0.00
9	-0.01	0.00	0.00	0.00	0.00	0.03	0.03	0.00	0.00
10	-0.11	-0.01	0.07	0.00	0.00	0.00	0.00	0.00	0.00
11	0.00	0.00	0.00	-0.01	-0.01	0.00	0.00	-0.01	-0.01
12	0.00	0.00	0.00	-0.05	-0.05	0.00	0.00	0.00	-0.01
13	0.00	0.00	0.00	0.00	-0.01	0.00	0.00	0.08	0.07
14	0.00	0.00	0.00	0.00	0.00	0.06	0.05	0.00	0.00
15	-0.07	-0.15	0.04	0.00	0.00	0.00	0.00	0.00	0.00
16	0.00	0.00	0.00	0.02	0.03	0.00	0.00	0.00	0.00
17	0.00	0.00	0.00	0.00	0.00	0.00	0.00	-0.01	-0.01
18	0.00	0.00	0.00	0.00	-0.01	0.00	0.00	0.00	0.00
19	0.00	0.00	0.00	0.00	0.00	-0.01	-0.01	0.00	0.00
20	-0.01	-0.02	-0.01	0.00	0.00	0.00	0.00	0.00	0.00

Table S9. Experimental (from CW-EPR) and calculated (CASSCF) zero field splitting parameters (*D*

and E in cm^{-1}) of Eu@C_{2n} molecules

	Eu@C_{74} (**3**)		Eu@C_{82}-C_s (**2**)		Eu@C_{84}-C_2 (**1**)		Eu@C_{80} (**3'**)	
	exp. EPR	calc. CASSCF	exp. EPR	calc. CASSCF	exp. EPR	calc. CASSCF	exp. EPR	calc. CASSCF
D	0.13	0.0363	0.29	0.0815	0.28	0.0812	0.275	0.0732
E	0.013	0.004	0.0025	0.0011	0.018	0.0151	0.0025	0.0186

Table S10. Relative energy (ΔE) of the spin levels of Kramers doublets (KD) in cm^{-1} for $^8S_{7/2}$ term of Eu@C_{74} (**3**).

KD	ΔE (CASSCF)
$M_S = 87.8\%\|\pm 1/2\rangle + 11\%\|\pm 3/2\rangle$	0.0000000
$M_S = 88.6\%\|\pm 3/2\rangle + 10.7\%\|\pm 1/2\rangle$	0.0973281
$M_S = 98.3\%\|\pm 5/2\rangle$	0.2369469
$M_S = 99.7\%\|\pm 7/2\rangle$	0.4500615

Table S11. Relative energy (ΔE) of the spin levels of Kramers doublets (KD) in cm^{-1} for $^8S_{7/2}$ term of Eu@C_{80} (**3'**).

KD	ΔE (CASSCF)
$M_S = 60.8\%\|\pm 1/2\rangle + 19.3\%\|\pm 3/2\rangle + 11.3\%\|\mp 1/2\rangle$	0.0000000

M_S = 72.4%$\vert\pm3/2\rangle$ + 19.5%$\vert\pm1/2\rangle$	0.3205068
M_S = 79.1%$\vert\pm5/2\rangle$ + 10.4%$\vert\mp5/2\rangle$	0.5887146
M_S = 85.5%$\vert\pm7/2\rangle$ + 12.7%$\vert\mp7/2\rangle$	0.9995833

Table S12. Relative energy (ΔE) of the spin levels of Kramers doublets (KD) in cm^{-1} for $^8S_{7/2}$ term of Eu@C_{82}-C_s (**2**).

KD	ΔE (CASSCF)
M_S=54.7%$\vert\pm1/2\rangle$ + 25.0%$\vert\mp1/2\rangle$ + 15.8%$\vert\pm3/2\rangle$	0.0000000
M_S = 63.7%$\vert\pm3/2\rangle$ + 16.9%$\vert\pm1/2\rangle$ + 14.7%$\vert\pm5/2\rangle$	0.1651449
M_S = 74.3%$\vert\pm5/2\rangle$ + 15.1%$\vert\pm3/2\rangle$ + 9.4%$\vert\pm7/2\rangle$	0.4918853
M_S = 89.1%$\vert\pm7/2\rangle$ + 9.4%$\vert\pm7/2\rangle$	0.9786101

Table S13. Relative energy (ΔE) of the spin levels of Kramers doublets (KD) in cm^{-1} for $^8S_{7/2}$ term of Eu@C_{84} (**1**).

KD	ΔE (CASSCF)
M_S=64%$\vert\pm1/2\rangle$ + 14.8%$\vert\pm3/2\rangle$ + 13.8%$\vert\mp1/2\rangle$	0.0000000

$M_S = 71.2\%\|\pm 3/2\rangle + 17.1\%\|\pm 1/2\rangle$	0.2883184
$M_S = 76.4\%\|\pm 5/2\rangle + 18.4\%\|\mp 5/2\rangle$	0.5852621
$M_S = 97.2\%\|\pm 7/2\rangle$	1.0572706

REFERENCE

1. L. Bao, Y. Li, P. Yu, W. Shen, P. Jin and X. Lu, *Angew. Chem. Int. Ed.*, 2020, **59**, 5259-5262.
2. Z. Slanina, F. Uhlík, L. Feng and L. Adamowicz, *Fullerenes, Nanotubes and Carbon Nanostructures*, 2016, **24**, 339-344.
3. L. Bao, P. Yu, C. Pan, W. Shen and X. Lu, *Chem Sci*, 2019, **10**, 2153-2158.
4. H. Yang, H. Jin, H. Zhen, Z. Wang, Z. Liu, C. M. Beavers, B. Q. Mercado, M. M. Olmstead and A. L. Balch, *J. Am. Chem. Soc.*, 2011, **133**, 6299-6306.
5. L. Bao, P. Yu, Y. Li, C. Pan, W. Shen, P. Jin, S. Liang and X. Lu, *Chem. Sci.*, 2019, **10**, 4945-4950.
6. Z. Hu, Y. Hao, Z. Slanina, Z. Gu, Z. Shi, F. Uhlik, Y. Zhao and L. Feng, *Inorg Chem*, 2015, **54**, 2103-2108.
7. H. Jin, H. Yang, M. Yu, Z. Liu, C. M. Beavers, M. M. Olmstead and A. L. Balch, *J Am Chem Soc*, 2012, **134**, 10933-10941.
8. H. Yang, H. Jin, X. Wang, Z. Liu, M. Yu, F. Zhao, B. Q. Mercado, M. M. Olmstead and A. L. Balch, *J Am Chem Soc*, 2012, **134**, 14127-14136.
9. N. C. Chang, J. B. Gruber, R. P. Leavitt and C. A. Morrison, *The Journal of Chemical Physics*, 1982, **76**, 3877-3889.
